# Is primary health care ready for artificial intelligence? What do primary health care stakeholders say?

**DOI:** 10.1186/s12911-022-01984-6

**Published:** 2022-09-09

**Authors:** Amanda L. Terry, Jacqueline K. Kueper, Ron Beleno, Judith Belle Brown, Sonny Cejic, Janet Dang, Daniel Leger, Scott McKay, Leslie Meredith, Andrew D. Pinto, Bridget L. Ryan, Moira Stewart, Merrick Zwarenstein, Daniel J. Lizotte

**Affiliations:** 1 Department of Epidemiology and Biostatistics, Schulich School of Medicine and Dentistry, Western University, 1151 Richmond Street, London, ON N6A 3K7 Canada; 2 Department of Family Medicine, Schulich School of Medicine and Dentistry, Western University, London, ON Canada; 3 Schulich Interfaculty Program in Public Health, Schulich School of Medicine and Dentistry, Western University, London, ON Canada; 4 Department of Computer Science, Faculty of Science, Western University, London, ON Canada; 5 Age-Well NCE, Toronto Rehabilitation Institute, Toronto, ON Canada; 6 London Middlesex Primary Care Alliance, London, ON Canada; 7 Thames Valley Family Health Team, London, ON Canada; 8 Upstream Lab, MAP/Centre for Urban Health Solutions, Li Ka Shing Knowledge Institute, Unity Health Toronto, Toronto, Canada; 9 Department of Family and Community Medicine, St. Michael’s Hospital, Toronto, Canada; 10 Department of Family and Community Medicine, Faculty of Medicine, University of Toronto, Toronto, Canada; 11 Dalla Lana School of Public Health, University of Toronto, Toronto, Canada

**Keywords:** Family medicine, Primary health care, Artificial intelligence, Qualitative research

## Abstract

**Background:**

Effective deployment of AI tools in primary health care requires the engagement of practitioners in the development and testing of these tools, and a match between the resulting AI tools and clinical/system needs in primary health care. To set the stage for these developments, we must gain a more in-depth understanding of the views of practitioners and decision-makers about the use of AI in primary health care. The objective of this study was to identify key issues regarding the use of AI tools in primary health care by exploring the views of primary health care and digital health stakeholders.

**Methods:**

This study utilized a descriptive qualitative approach, including thematic data analysis. Fourteen in-depth interviews were conducted with primary health care and digital health stakeholders in Ontario. NVivo software was utilized in the coding of the interviews.

**Results:**

Five main interconnected themes emerged: (1) Mismatch Between Envisioned Uses and Current Reality—denoting the importance of potential applications of AI in primary health care practice, with a recognition of the current reality characterized by a lack of available tools; (2) Mechanics of AI Don’t Matter: Just Another Tool in the Toolbox– reflecting an interest in what value AI tools could bring to practice, rather than concern with the mechanics of the AI tools themselves; (3) AI in Practice: A Double-Edged Sword—the possible benefits of AI use in primary health care contrasted with fundamental concern about the possible threats posed by AI in terms of clinical skills and capacity, mistakes, and loss of control; (4) The Non-Starters: A Guarded Stance Regarding AI Adoption in Primary Health Care—broader concerns centred on the ethical, legal, and social implications of AI use in primary health care; and (5) Necessary Elements: Facilitators of AI in Primary Health Care—elements required to support the uptake of AI tools, including co-creation, availability and use of high quality data, and the need for evaluation.

**Conclusion:**

The use of AI in primary health care may have a positive impact, but many factors need to be considered regarding its implementation. This study may help to inform the development and deployment of AI tools in primary health care.

**Supplementary Information:**

The online version contains supplementary material available at 10.1186/s12911-022-01984-6.

## Background

Artificial Intelligence or AI is increasingly being recognized as having potential importance to the provision of primary health care [1]. Our definition of AI is as follows: “The field of AI is broad and rapidly expanding. The field is centred on how computers might be able to perform humanlike “intelligent tasks,” such as summarizing large amounts of information or making inferences about a situation”[2]. The ways in which AI may be useful to primary health care have been described [1, 2], and include predicting pre-specified outcomes, exploring and describing data, and providing recommendations or decision support. Current examples of AI tools for primary health care are varied and include those focused on risk prediction [3–6], workforce assessment [7], and extracting information from narrative EMR data [8, 9]. Within the broader health care context, uses of AI in health care continue to grow [10] and include the use of conversational agents e.g., chatbots [11], radiology products [12], and predicting healthcare associated infections [13]. The rising prominence of AI in health care is reflected by the increasing production of published [14, 15], and grey literature [16–19] related to this topic.

In this paper, we focus on AI in primary health care specifically. There are unique features of primary health care including: first contact care provision; a patient population attached to specific practices; care that is provided on a long-term basis; and primary health care practitioners who coordinate care across the health system. These features mean that the nature of the care provided, and the data utilized in the provision of primary health care, are different than in other areas of healthcare. Therefore, AI tools intended for use in primary health care need to be tailored for this setting. Despite the emergence of AI tools intended for use in primary health care, their adoption has been limited [20, 21]. Significant gaps in knowledge regarding the development, implementation, and evaluation of AI in primary health care have been identified [6, 16, 20, 21]. Specifically, primary health care AI tools are being developed and assessed without the inclusion of the intended end-users of most of these tools, i.e., primary health care practitioners [20, 21]. Effective deployment of AI tools in primary health care requires the engagement of practitioners in the development and testing of these tools, and a match between the resulting AI tools and clinical/system needs in primary health care. To set the stage for these developments, we must gain a more in-depth understanding of the views of practitioners and decision-makers about the use of AI in primary health care.

This paper reports the findings from one of a series of studies designed to explore these issues [22]. This qualitative study explored the uses of AI in primary health care. Our objective was to identify key issues regarding the use of AI tools in primary health care by exploring the views of primary health care and digital health stakeholders. We intentionally sought to gain a holistic understanding of these issues by engaging different primary health care stakeholders who bring unique perspectives.

## Methods

We used a descriptive qualitative approach in this study. With this approach, the researcher seeks to “discover the who, what and where of events or experiences” [23, 24]. This study received ethics approval from The University of Western Ontario’s Review Board for Health Sciences Research Involving Human Subjects (Project ID #116,208) and all methods were carried out in accordance with the relevant guidelines from the Review Board.

### Setting and participant recruitment

This study took place in the province of Ontario, Canada from September 2020 to February 2021. Participants were recruited from a purposive sample of primary health care and digital health stakeholders generated through investigator team member existing networks and a search of relevant organizational websites and publications. Representation was sought from across stakeholder roles, including primary health care practitioners, decision-makers, and researchers with expertise or interest in digital health/AI in primary health care, from different geographic locations across the province of Ontario. A letter of information and consent that explained the study aims and outlined the request for participation was sent by email to this group of potential participants. If there was no response to the initial recruitment email, two email reminders approximately one week apart were sent. No further contact was made if there was no response to the second reminder. Several study participants were known to researchers ALT, DJL, and JK in a professional capacity.

### Data collection

Twenty-six individuals were approached to participate in the study, and fourteen agreed. Four individual semi-structured interviews were conducted by study investigator ALT, five by JK and five by DJL. Both ALT (PhD Epidemiology) and DJL (PhD Computer Science) are Associate Professors, and JK is a PhD Candidate (Epidemiology and Computer Science) at Western University. ALT and DJL are experienced primary health care researchers. All interviewers have apprenticed with expert qualitative researchers. All interviews took place using Zoom conferencing system or by telephone; audio-only recordings of the interviews were made, and the recordings were transcribed verbatim. Interview guide questions focused on key challenges regarding primary health care provision, the possibilities of the use of AI in primary health, and what we should be trying to achieve with AI in primary health care. We shared the following broad definition of AI during the interviews: “When we say AI, we mean the use of tools that automate tasks that ordinarily would require human intelligence like when doing data analyses, trying to process lots of test, and providing decision support for prognoses and diagnoses”; we did not instruct the participants to focus or restrict their responses to this definition of AI. As this study took place during the COVID-19 pandemic, we included questions that related to the pandemic, particularly with respect to primary health care. Interviews were an average of thirty-five minutes in length.

### Data analysis

Our approach to the analysis was iterative and interpretive. Three investigators (JK, DJL, ALT) coded the initial set of interview transcripts independently, then came together to create a coding template and to compare and discuss the results of this independent coding. Adjustments were made to the coding template as the interviews proceeded. This pattern of analysis was followed for the remainder of the interviews. NVivo software (QSR International Pty. Ltd. 2018 Version 12) was utilized in the coding of the interviews [25]. Techniques of immersion and crystallization were employed in the analysis process [26]. The investigator team met weekly for the purposes of data analysis and synthesis of the themes. Sufficient data were obtained to support theme saturation [27].

### Credibility and trustworthiness of the data

The team undertook several steps to enhance the trustworthiness and credibility of the data. We used both individual and team analysis techniques. First, interview transcripts were reviewed for accuracy by team members. Second, each team member who conducted interviews took field notes after each interview; these notes were reviewed and discussed at team meetings. This process supported adaptation of the interview questions and reflective discussions about the concepts and themes emerging from the interviews [28]. Reflexivity of this nature is an important component of qualitative research [28].

## Results

Fourteen primary health care and digital health stakeholders participated in in-depth interviews. These participants had dual roles in some cases and included: four decision makers; two decision maker/researchers; five primary health care practitioner/researchers; and three researchers. Participants had varying levels of exposure to AI use in primary health care but all had interest and/or knowledge about AI. Ten of the participants had research interests in AI/digital health in primary health care. Decision-maker participants worked in health-related areas of government, health systems and data/health information science. Participants who were primary health care practitioners were interested in AI use but many did not have had specific exposure to the direct use of AI tools in clinical practice. These participants came from 13 organizations located in four cities in Ontario. Interviews took place between October 2020 and February 2021.

From our analysis of the data five overarching themes, reflecting key issues in the use of AI in primary health care, emerged: Mismatch Between Envisioned Uses of AI and Current Reality; Mechanics of AI Don’t Matter: Just Another Tool in the Toolbox; AI in Practice: A Double-Edged Sword; The Non-Starters: A Guarded Stance Regarding AI Adoption in Primary Health Care; and Necessary Elements: Facilitators of AI in Primary Health Care. These themes were not mutually exclusive. Indeed, participants often held overlapping views in these areas, highlighting the interconnectedness of the themes. Overall, participants often shared their thoughts about health information technology in general and linked these ideas more specifically to AI use in primary health care. They also commented on current challenges in primary health care, including the influence of the COVID-19 pandemic in relation to primary health care and AI. Please see Table 1 for summary points of the study results.

**Table 1:** Summary points regarding the use of artificial intelligence in primary health care

Summary points regarding the use of artificial intelligence in primary health care
• Participants viewed AI with a guarded but hopeful stance
• AI use in primary health care is in an early stage of maturity
• AI tools relevant to the needs of primary health care practitioners need to be developed
• The role of AI in primary care health and clinical encounters needs to be determined
• Threats regarding AI in terms of clinical skills and capacity included: concerns over impact on clinical skills, introduction of errors, and loss of control in decision-making
• Ethical, legal, and social implication of AI use included: medical-legal concerns and potential biases in AI tool and equity considerations; lack of transparency related to black-box AI creation; loss of control over data; and privacy and security of data
• Necessary foundational elements to support the uptake of AI tools included: co-creation of AI tools, availability and use of high-quality data, rigorous evaluation of AI tools and/or a strong evidence base

### Overarching themes

#### Mismatch between envisioned uses of ai and current reality

Multiple uses of AI in primary health care were envisioned including those focused on clinical practice, on patient/caregivers, and on analyzing data. Applications to support practice included examples such as decision support and routine task completion. AI was cited as being useful for patient self-management and preventive care, as one participant indicated:

*“…they [patients] want to know why ...their hemoglobin is off by one point… actually, AI might be a solution for that, right? If it can give them answers to things they want to know. Again, back to that self-care, chronic disease management, I think as long as it’s as authoritative or as peer reviewed as the current literature allows or suggests.”* Participant 117

Participants noted AI tools could be used for analyzing practice data for the purposes of practitioner feedback and managing patient populations, and in the analysis of existing evidence to support patient care:

*“If a general practitioner has patient data from a person for many, many years and predictive analytics can analyze that data and say ‘OK, over the past 10 years this person’s blood pressure has been steadily increasing, maybe we should act on this now’ I think that will be a really useful tool for healthcare providers to have.”* Participant 142

This vision of these potential uses of AI was juxtaposed with the reality that there are very few AI tools that have been applied in the primary health care setting, despite their ubiquity in daily life.

One participant noted:

*“Right now I don’t know anyone that’s...really using an AI tool beyond some gimmick… I don’t really know anyone that’s using something very sensible… we just tend to use apps on our phone that are just very convenient point of reference tools…”* Participant 110

This was coupled with the sense that companies producing AI tools were poised to take advantage of this gap. *“Those companies are already here. And they’re already going to be putting stuff into place before primary health care can even lift its head up to realize that it’s here”* Participant 141.

Overall, participants noted a need to capitalize on the use of AI in primary health care, as one participant stated: *“AI is – like, our only saving grace for many people… we’ve got to really take advantage of this technology”.* Participant 143.

In summary, participants identified the importance of applications of AI in primary health care practice (including decision support, routine task completion, patient self-management, analyzing practice data, and analysis of existing evidence), while recognizing the current reality posed by a lack of available tools. AI was viewed as coming closer on the horizon however, producing a sense of urgency in ensuring these tools would be explored in terms of application in clinical practice.

#### Mechanics of AI don’t matter: just another tool in the toolbox

Participants were not concerned with the mechanics of AI, rather they were most interested in the value that AI tools could bring to practice. This practical view also extended to the role they saw AI playing in terms of direct patient care, where existing relationships and human interaction would not be superceded by AI.

Fundamentally, practitioners saw the value in AI tools aiding aspects of patient education and care that didn’t require a clinician (such as the provision of patient education materials), regardless of the nature of the tool:

*“I don’t have to be Dr. Do It All. So, if we can download some of that care or some of that patient education or chronic disease management to an AI system and whether it’s a chatbot or whether it’s materials coming to the patient or maybe a flag of their lab results if they need to reassess their diabetic care, etcetera, I think that’s got potential.”* Participant 117

This perspective on AI tools was aligned with participant views about the place of AI in terms of patient care:

*“Yeah, AI will never be able to do that… reading the body language, reading emotions, the longstanding relationship you have with that patient. So that has to be seen as always being a peg above what AI could do”.* Participant 143

Within this theme, there was disinterest in the inner workings of AI tools—rather, participants were focused on what AI could do for them. Participants felt that AI would never be able to perform the complex interactions necessary for effective patient care, nor replace the critical role of the patient-practitioner relationship. Thus, AI was viewed in a utilitarian manner, placed in the background in terms of patient care, with a clear focus on its practical value.

#### AI in practice: a double-edged sword

There were strong views expressed about the possible benefits of AI in primary health care, and participants saw opportunities associated with its use, for example:

*“You know if we think about how many prescriptions we write every day… how much data we’re feeding into a system, test results that are coming... I’ve got to manually process that stuff right now and its super time-consuming, laborious and unfortunately there’s no better way to do it, so I’ve got to do it...it feels like it takes up an hour or two of every day and if I had something, a machine, that could do that for me in an accurate way and alert me when there’s something that I need to look at, like it’s red-flagging things in real time then that would be a huge breakthrough because then I could identify patient safety issues as they happen”.* Participant 110

However, they also expressed fundamental concern about the possible threats posed by AI in terms of clinical skills and capacity. One participant noted: *“… there is that downside as well if we offload everything to the machine if it were possible, we would also become worse in another way. We would lose our intellectual capacity…”* Participant 110. Other participants noted concerns about AI making mistakes and adding to the stress experienced by practitioners rather than helping to ameliorate workload: *“Now, something that could come into place and relieve some of that burden, that’s great. But, if it could add to that stress at all, what if the AI gets it wrong?”* Participant 122. Finally, there was a fear of a loss of control, with AI taking over decision making rather than being a support for practitioners: “*I'm much more comfortable with AI as an assistive tool, rather than making decisions on its own.”* Participant 143.

Thus, an essential tension exists between the possible benefits of AI and the possible risks, particularly in terms of clinical skills and practice. This was further revealed in the next theme that captured the broader context.

#### The non-starters: a guarded stance regarding AI adoption in primary health care

Along with concerns about professional skills and capacity, participants expressed broader concerns centred on the ethical, legal, and social implications of AI use in primary health care.

First were possible consequence of AI use. Within this theme, participants noted medical-legal implications:

*“I spend a lot of time going through refilling prescriptions… just looking at, “Oh, there’s John, his blood pressure’s good …three more months or a years’ supply.” Similar for maybe diabetic refills…. could, in the back end, the AI read maybe an NLP [natural language processing], to look at those indicators of blood pressure, lab parameters, and have some intuition, this would be fine, to release that. …What would be the medical legal implications of that, obviously?* Participant 117

Participants expressed concern over potential biases in AI tools, and equity considerations:

*“I think another big challenge with AI is the sort of algorithmic bias that can come with the development of the AI systems, another challenge to think about [is] the actual development of the AI that’s being used and ensuring that it’s not biased and it’s really promoting an equitable system”* (Participant 142).

Panch et al. have defined algorithmic bias in the context of AI and health as: “the instances when the application of an algorithm compounds existing inequities in socioeconomic status, race, ethnic background, religion, gender, disability or sexual orientation to amplify them and adversely impact inequities in health systems” [29]. Ethical elements also came into play: “…the business model behind these technologies is where I think the most important ethics conversation is to be had at the moment” (Participant 122). Participants noted the level of risk in the use of these tools in the provision of health care:

*“…deployment of the algorithm, would it have led to better outcomes? And, importantly, did it present any risks?... – all clinicians are socialized into this ethic of, “The most important thing is that I protect my patients.” And so, that mentality, it pervades the healthcare professional culture, and especially medicine”* Participant 122

Second, AI tools are typically created by for-profit companies. The proprietary nature of these tools means that black box AI creation often exists, where there is little transparency in terms of how the tools actually work and what data are being used for AI applications. Participants expressed mistrust about this situation:

*“…as we move into these AI platforms, the concern I have is a lot of the algorithms are proprietary and [people] were very secretive in how they’re organizing these - what’s on the back end and how trusting can I be of what it’s telling me?”* Participant 117

To allay this fear, participants wanted detailed and clear explanations:

*“I think that they [patients and practitioners] [need to] know what goes into the black box. I’d want to make [sure] that it was actually explained and articulated how the end result came about. I just wouldn’t want any assumptions to be made. Sometimes at the system level decisions are made that may not take into consideration all the different aspects. I don’t think that would happen but having all of the different pieces put together is important; the contextual pieces.”* Participant 111

Third, there was sense that the use of AI could result in a loss of control over how data could be used, with concerns about data being used for profit or in unethical ways, resulting in the need to carefully protect these data:

*“[what] we need to add is a way to make sure that personal health information is protected and that we don't step outside social licence.” “...how can we make sure that the companies don’t just profit from it, and how do we make sure that people aren’t disadvantaged, and do people really understand that their data are being used.” Participant 108*

As another participant noted: *“the other part is [what is] behind the – maybe the dirty side of AI, which is the monetization of big data.”* Participant 117 This was coupled with concerns over the privacy and security of data:

*“...and same with the privacy concerns, there are already data leaks happening all the time and I think with more and more patient data being stored on an AI system or a computer system, it’ll just be even more important to safeguard that data”.* Participant 142

Participants were guarded in their views about AI; they could envision AI tools and their uses but were concerned about unforeseen consequences of AI use. These concerns were viewed as non-starters, i.e., if they were not addressed, then primary health care would not be ready to accept and implement AI, regardless of how the AI tools performed.

#### Necessary elements: facilitators of AI in primary health care

Participants noted that foundational elements were required to support the uptake of AI tools and other technological innovations. These elements included the importance of co-creation:

*“…that co-design piece of having the end users—so most likely nurses, doctors, nurse practitioners, anyone who’s going to be using the technology really needs to be involved in the development and co-design of the technology from the beginning. …right now, with other types of technology, it will be this tech company that’s developing this great system and they only consult the end users when it’s finished and then it’s almost too late to kind of incorporate things that really should’ve been included from the beginning. So that is something that would be really important to emphasise and could help with a successful implementation of any AI technology as well.”* (Participant 142)

A second element was the availability and use of high-quality data, as one participant noted*: “I think the biggest challenge…is around data standardization. The actual existence of that data, the interoperability of that data, the ability for that data to be machine interpretable”.* (Participant 128).

Finally, participants noted that there needed to be certainty that the AI tools being deployed had been evaluated or were based on good evidence, particularly in relation to clinical care:

*“If there’s no RCT [randomized control trial] then I don’t know that it’s safe, and I don’t know that it’s effective, and I’m not going to deploy it in relation to my clinical decision making. Maybe practice management is different, but [for] any kind of clinical intervention that would involve AI, the lack of a traditional evidence base on it presents major challenges to moving forward.”* (Participant 122)

These elements were viewed as foundational to any intervention or innovation with application in primary health care practice. The need for co-creation, high quality data, and rigorous evaluation of AI were viewed as facilitators of the ultimate uptake of AI in primary health care.

#### COVID-19 pandemic

Finally, reflecting on their experiences with COVID-19, participants shared common views about changes in patient care such as the shift to virtual care. They also described the inherent system change and the use of new tools, especially technology-associated tools, as a result of the disruptive influence of the pandemic:

*So this flood* [shift in health care as a result of COVID-19] *changed a lot of things in the way that people had to step back and say “OK, the way I was doing it before, that’s gone now, and I need to do things differently.” So I think there’s a huge opportunity created by that. My view is that it will be largely a positive opportunity because there’s a recognition that when people start picking up these tools, they do like them and we’re absolutely seeing that.* (Participant 128)

There was an enhanced urgency around system change and the use of these tools; these views related to all five of the overarching major themes identified. Please see Additional file 1 for further participant quotes supporting the themes described in the results section.

## Discussion

This study illuminated key tensions regarding the use of AI in primary health care. While participants could see the potential of AI in the primary health care setting, they also expressed significant concerns. As a whole, the five themes that emerged represent a guarded but hopeful stance regarding the use of AI in primary health care. These themes were interrelated, with overlapping elements, and illustrate several multifaceted considerations regarding the implementation of AI in primary health care including: the development of AI tools relevant to the needs of primary health care practitioners; determining the role of AI in clinical encounters; reconciling threats to clinical skills and capacity; addressing broader concerns regarding consequences of AI use, lack of transparency, and control over data; and ensuring foundational elements of co-creation, high quality data, and evaluation of AI tools are in place. Please see Table 2 for key messages from this study. Although the existing literature is relatively sparse, research from other jurisdictions exploring perceptions of AI among healthcare stakeholders echo these findings [30–33].

**Table 2:** Key messages

Key messages
• Users of AI tools need be engaged in co-design processes; the gravity of this situation is further heightened by the black box creation of AI
• High quality, standardized data are essential to support the functioning of AI tools
• A strong evidence base, created from systematic assessments of AI tools in the primary health care setting is required

Participants in this study could envision many uses of AI in primary health care and noted an urgent need to manage and capitalize on this technology. This was juxtaposed against a lack of AI tools being implemented in current practice, reflecting an early stage of maturity of AI use in primary health care. While AI tools continue to be produced, the lack of widespread adoption in practice is likely because there are significant gaps in the research evidence around the development and implementation of AI in primary health care, in particular the lack of assessment and testing of AI in actual practice; similar conclusions have been reached about the latter finding in terms of the broader health care setting [34]. There are also overarching concerns about AI use in health care highlighted in this study and others [35–38].

In this study, participants had a utilitarian view of AI, where the focus was on the value that the tools could provide in terms of patient care. AI was placed in the background, never replacing the complex tasks of the clinician, nor subverting the patient-physician relationship; these findings parallel those from other countries [32, 33]. Similarly, participants in a study of patient portals identified the centrality of the patient-physician relationship and noted that portals should be considered as simply another tool that would be part of their health care [39]. We do know that the use of other technologies such as electronic medical records, can be disruptive to patient-practitioner encounters [40, 41]. It remains to be seen what the use of AI will mean for patient-practitioner interactions and how we might mitigate these impacts, or maximize the benefits.

While participants described many possible opportunities associated with AI use, they also expressed strong concerns about the threat AI could pose to clinical skills and capacity. Similarly, in an international Delphi study, respondents identified both benefit and risks to the use of AI in primary health care [42]. The relative immaturity of AI tools and their use in primary health care results in the need for strong oversight and guidance for the “ethical and rigorous development of AI applications so that they will be safe and effective in the workplace” [42]. Trust is a critical component in the interaction of practitioners and AI [43, 44], and participants expressed a definite lack of trust in this technology. Along with their specific concerns about AI use in practice, participants in this study also expressed broader concerns associated with consequences of AI use, lack of transparency in AI creation, and a potential loss of control over data. These wider ethical, legal, and social implications of AI use in primary health care have led to proposed regulations about AI use more broadly [45], as well as recommendations for ethical use of electronic health record data and AI in primary health care [46].

In this study, participants noted the critical importance of three elements that could help facilitate the uptake of AI. First was a clear message that the users of this technology need be engaged in co-design processes. Few examples of this kind of engagement exist [20, 21, 47]. There are important parallels here to the implementation of electronic medical records in primary health care, where there was a distinct lack of end-user involvement in the creation and assessment of these systems. This situation is being repeated in AI development and implementation [20, 21]. Leading experts in this field have therefore called for the involvement of primary health care practitioners in AI development and assessment [48]. The gravity of this situation is further heightened by the black box creation of AI, resulting in an even more opaque technology than electronic medical record software, with greater promise but also potentially greater pitfalls. Second, participants noted the importance of high quality, standardized data to support the functioning of AI tools; a critical aspect of the use of AI that has also been identified by others [49, 50]. Third is the need for a strong evidence base, created from systematic assessments of these tools in the primary health care setting [17, 20, 21]. Thus, there is a gap in our understanding about how to best utilize AI in primary health care settings as this is an emerging area where there is a lack of high-quality research evidence.

Given the current early stage of the adoption of AI tools in primary health care, the participants in this study were describing a future state based on their current knowledge of AI, rather than its actual use in practice. Although we did not study the actual use of AI, there are aspects of the themes that emerged that align with existing technology acceptance models [51, 52]; extensions of these models for health care AI exist [35], however they are at an early stage of development. The Unified Technology Acceptance Model posits that four constructs are determinants of technology acceptance and use behaviour: “performance expectancy, effort expectancy, social influence, and facilitating conditions” [52]. In this study, one could consider three of these constructs being relevant: The first is performance expectancy—“…the degree to which an individual believes that using the system will help him or her to attain gains in job performance” which is aligned with our first theme, uses that participants envisioned for AI in primary health care and the prospective value of AI tools [52]. Second is effort expectancy—“the degree of ease associated with the use of the system”; this links with the “non-starters” theme of this study where ultimately even if the AI tool itself were easy to use, concerns over its use create a significant level of complexity [52]. Third is facilitating conditions which are defined as “the degree to which an individual believes that an organizational and technical infrastructure exist to support the use of the system” [52]; this construct relates to participant’s concerns about control over AI and how this technology would fit with existing ways of working [52].

Other examples of frameworks that focus not just on technology acceptance but also on the full scope of implementation and evaluation of health information innovations in clinical settings exist [53, 54]. These models pay careful attention to the socio-technical aspects of health information technology adoption, use, and evaluation, including: “technical and nontechnical factors, such as workflow and organizational issues” [55]. These factors are inter-related and are essential to understanding the complex picture presented by information technology innovations in health care. Many of the key issues regarding AI use in primary health care presented in this study, including serious concerns over introduction of errors, loss of control, and strong reactions to a possible loss of intellectual capacity, mirror well-known socio-technical factors found in classic studies in other health care settings [56]. There are many other examples in the primary health care setting where studies of information technology innovations have also identified socio-technical issues as important factors impacting the uptake and use of these interventions [57–59]. Many of the challenges identified in these studies are congruent with those identified in the present study, including the nature of the information technology tools, their use in patient care, and ethical, legal, and social issues. Participants in the present study expressed concerns that the use of AI could undermine clinical skills and capacity particularly because the possible uses of AI in clinical practice are very broad; similar issues have been raised previously regarding other health information technologies but were amplified by AI’s breadth of potential application. Newer concerns related to AI use include algorithmic bias and black box AI creation. This lack of transparency around AI creation is particularly important given that, for the most part, AI tools operate in the background of a computerized environment. Not being able to clearly identify how these tools are created and operationalized poses a significant issue in terms of practitioners’ trust in this technology; this will have a subsequent impact on the eventual use of AI tools in practice.

The main limitation of this study is that we had a relatively small number of participants (n = 14); therefore, the study findings reflect the perspectives of this group of participants and may not fully capture stakeholder views regarding AI in primary health care. Despite this, we were able to explore the perspectives of a group of primary health care and digital health stakeholders across Ontario who held different professional roles. Key themes identified in this study illuminate these perspectives with respect to the use of AI in the primary health care setting.

The findings of this study illustrate that primary health care’s readiness for AI is contingent on the resolution of several fundamental aspect of this technology. Participants could see the value in the use of AI tools, but this promise was overshadowed by serious concerns about the nature of the tools, their use in patient care, and ethical, legal, and social issues.

## Conclusion

The use of AI in primary health care may have a positive impact in the future, but many factors need to be considered regarding its implementation. New technology is often implemented using a top-down approach to fix a problem or meet a specific need and often the end user can be forgotten in its development and implementation processes. This project revealed that the implementation of AI may have unique properties that allow a bottom-up approach. This is because AI needs to relate more to its end user—how users think, how they interact, and how they make decisions as well as consideration of the patient experience. Overall, the findings of this study may help to inform the development and deployment of AI tools in primary health care.

## Supplementary Information

**Additional file 1.** Supplement: Additional Quotes by Theme.

## Data Availability

The datasets generated and/or analyzed during the current study are not publicly available because they contain information that could compromise research participant privacy but are available from the corresponding author on reasonable request.

## Abbreviation

AI: Artificial intelligence
