# Supplementary material for: Is primary health care ready for artificial intelligence? What do primary health care stakeholders say?
Source: BMC Med Inform Decis Mak. 2022 Sep 9;22:237. doi: 10.1186/s12911-022-01984-6 (PMC9461192; doi:10.1186/s12911-022-01984-6)
Supplement: Supplementary file 1 — Additional file 1. Supplement: Additional Quotes by Theme. [file 12911_2022_1984_MOESM1_ESM.docx]

**Supplement: Additional Quotes by Theme**

**Theme 1** Mismatch Between Envisioned Uses and Current Reality – denoting the importance of potential applications of AI in primary health care practice, with a recognition of the current reality characterized by a lack of available tools:

“I’ve heard and read about, you know, the NLP [natural language processing] that can record your interview …with the patient or consultation with the patient … how great that would be… It’s not that far off and I’m sure there’s probably some good models out there that are being used… that’s the dream, right?” (Participant 110)

“I think that the big challenge for using the artificial intelligence in primary care is more of an infrastructure than many other things”. (Participant 136)

**Theme 2:** Mechanics of AI Don’t Matter: Just Another Tool in the Toolbox– reflecting an interest in what value AI tools could bring to practice, rather than concern with the mechanics of the AI tools themselves:

“So in areas of the world for example where there isn’t a primary care system, or there isn’t a strong primary care system, can providers enter into a database a question “I’m seeing a patient right now with A, B and C” and then when I press that button I can see, I can go through that database and give me a response. “Given what you’ve said here in your question, these are the best matches for outcome.” So maybe it’s a chat bot, you’re actually talking. Talking to the system or inputting in the system and you’re getting this type of response”. (Participant 135)

“..the big challenge that I've identified is sort of a workforce that's disappeared. So we need to think up different ways of doing things obviously and while I would fight you to the death if you tried to say to me what I'm just going to say to you, which is that a machine of any kind can interact well enough with a human being to replace their family doctor… one of the big things about family medicine is that relationship and that longitudinality. Now certainly the machine could pick up things over time and would probably be better at remembering a lot of the nuances from 20 years ago than I am, but I think our technology would need to be really improved a lot.” (Participant 138).

**Theme 3:** AI in Practice: A Double-Edged Sword – the possible benefits of AI use in primary health care contrasted with fundamental concern about the possible threats posed by AI in terms of clinical skills and capacity, mistakes, and loss of control:

“I feel like when AI is making those kinds of contributions where it’s actually identifying things that humans would not identify. And that’s in front of people; they can see it, they can say ‘You know what, I just missed a breast cancer diagnosis and this tool caught it.’ That’s when things start to become really interesting”. (Participant 128)

“…As we get further out, you know, will there come a point where it’s [AI] giving me a diagnostic or a solution and I don’t question it because it’s dumbed me down?” (Participant 117)

“…maybe it’s [AI] giving me information based on… the data fed into it if there was an uptick on aortic stenosis or rheumatoid valve disease, etcetera and it’s erroneously cautioning me or giving me a flag that you really wouldn’t worry about otherwise.” (Participant 117)

“So we just need to look at this topic completely different, in terms of its barriers [rather] than your traditional EMR [electronic medical record], CPOE [computerized provider order entry] and adoption. Because it’s not this thing you’re touching. It just sits in the interstitial spaces between physical and digital that now is going to change you and in subtle ways (Participant 141).

**Theme 4** The Non-Starters: A Guarded Stance Regarding AI Adoption in Primary Health Care - broader concerns centred on the ethical, legal, and social implications of AI use in primary health care:

“So there’s a lot of trust and, you know, the actual programmers, most by and large don’t go to medical school themselves. There's a lot of interdisciplinary sort of interaction that has to occur, because when they're developing the models, the ethics that we adhere to as part of practicing clinicians, must be embedded into – right from the get-go, even before actually writing the code, in the conception of the model itself”. (Participant 143)

“There’s a lot of oversight that needs to occur at that level, before it’s even deployed… Because what AI will do is, it will just spit out whatever you put in. You know, the data in, data out thing is never more true for AI, and the models need to be calibrated at that programmatic level to make sure that it’s not exacerbating existing sort of negative issues that are embedded in our healthcare system, you know” (Participant 143)

“I’ve really come to realize that we have the ability with these systems because they can look at things that human cognition can’t to really spot or identify things where people’s equity is being compromised, either intentionally or unintentionally, and hopefully create better systems for people to live in. And here comes the half-empty side of me, I don’t think that’s going to happen. I really just don’t..” (Participant 141)

“And same with the privacy concerns, you know, there’s already data leaks happening all the time and I think with more and more patient data being stored on an AI system or a computer system, it’ll just be even more important to safeguard that data”. (Participant 142)

**Theme 5** Necessary Elements: Facilitators of AI in Primary Health Care - elements required to support the uptake of AI tools, including co-creation, availability and use of high quality data, and the need for evaluation

“…Engaging people in the process of building tools would be super helpful so they actually see what’s going on and have some feedback and contribution to it”. (Participant 111)

“I think the biggest challenge as I’m sure you are well aware of, is around data standardization. The actual existence of that data, the interoperability of that data, the ability for that data to be machine interpretable” (Participant 128)

“I suppose with physicians, evidence that something works won’t necessarily convince them to do it, but the lack of evidence about something will convince them not to do it”. (Participant 122)
